# Effects of High-Pressure Processing, UV-C Irradiation and Thermoultrasonication on Donor Human Milk Safety and Quality

**DOI:** 10.3389/fped.2022.828448

**Published:** 2022-03-21

**Authors:** Eva Kontopodi, Bernd Stahl, Johannes B. van Goudoever, Sjef Boeren, Rian A. H. Timmermans, Heidy M. W. den Besten, Ruurd M. Van Elburg, Kasper Hettinga

**Affiliations:** ^1^Department of Pediatrics, Emma Children’s Hospital, Amsterdam UMC, University of Amsterdam, Amsterdam, Netherlands; ^2^Food Quality and Design Group, Wageningen University & Research, Wageningen, Netherlands; ^3^Department of Chemical Biology & Drug Discovery, Utrecht Institute for Pharmaceutical Sciences, Utrecht University, Utrecht, Netherlands; ^4^Danone Nutricia Research, Utrecht, Netherlands; ^5^Laboratory of Biochemistry, Wageningen University and Research, Wageningen, Netherlands; ^6^Wageningen Food & Biobased Research, Wageningen University and Research, Wageningen, Netherlands; ^7^Food Microbiology, Wageningen University and Research, Wageningen, Netherlands

**Keywords:** donor human milk, non-thermal processing, bacteria inactivation, bacteriostatic properties, proteomics, antimicrobial proteins

## Abstract

Holder pasteurization (HoP) is the current recommended treatment for donor human milk. Although this method inactivates microbial contaminants, it also negatively affects various milk components. High-pressure processing (HPP, 400, 500, and 600 MPa), ultraviolet-C irradiation (UV-C, 2,430, 3,645, and 4,863 J/L) and thermoultrasonication (TUS, 1,080 and 1,620 kJ/L) were investigated as alternatives to thermal pasteurization (HoP). We assessed the effects of these methods on microbiological safety, and on concentration and functionality of immunoglobulin A, lactoferrin, lysozyme and bile salt-stimulated lipase, with LC-MS/MS-based proteomics and activity assays. HoP, HPP, TUS, and UV-C at 4863 J/L, achieved >5-log_10_ microbial reduction. Native protein levels and functionality showed the highest reduction following HoP, while no significant reduction was found after less intense HPP and all UV-C treatments. Immunoglobulin A, lactoferrin, and lysozyme contents were also preserved after low intensity TUS, but bile salt-stimulated lipase activity was significantly reduced. This study demonstrated that HPP and UV-C may be considered as suitable alternatives to HoP, since they were able to ensure sufficient microbial inactivation while at the same time better preserving the bioactive components of donor human milk. In summary, our results provide valuable insights regarding the evaluation and selection of suitable processing methods for donor human milk treatment, which may replace HoP in the future.

## Introduction

Human milk (HM) is universally identified as the normative standard for infant nutrition, due to its unique nutritional composition and bioactive components such as immunoactive proteins, hormones, and growth factors, that facilitate proper infant growth and development (1). An essential component of HM known for its bioactive function is the HM proteome, which is comprised of a wide array of proteins, glycoproteins, enzymes, and endogenous peptides (2). For example, HM exerts bacteriostatic activity, a function largely ascribed to the presence of bioactive proteins, such as immunoglobulin A (IgA), lactoferrin (LTF), and lysozyme (LYZ), due to their high abundance in HM [(3, 4)]. More specifically, IgA protects the infant from invasive pathogens, LTF inhibits the growth of iron-dependent pathogens and LYZ lyses the proteoglycan matrix of the cell walls in Gram-positive bacteria. In addition, a synergistic effect of LYZ and LTF is suggested against Gram-negative bacteria (4–6)].

In case mother’s own milk is unavailable, donor human milk (DHM) is the best alternative and it should be provided by established human milk banks that enforce all necessary actions to guarantee its safety (7, 8). Human milk banking guidelines recommend holder pasteurization (HoP) for the elimination of possible life-threatening pathogens in DHM (9). This method is performed by heating DHM for 30 min at a temperature of 62.5°C, followed by a rapid cooling down to <10°C (10).

Even though HoP achieves the 5-log_10_ reduction of vegetative bacterial cells required by all human milk banking guidelines, it also leads to the degradation of key DHM bioactive components (9). After HoP, a significant reduction has been reported in the concentration and activity of IgA, LTF, and LYZ, as well as in several enzymes, hormones, cytokines, and growth factors (10, 11). In addition, HoP completely inactivates bile salt-stimulated lipase (BSSL), a heat-labile enzyme that facilitates fat absorption and enhances lipid metabolism (11). It is thus possible that BSSL inactivation through HoP may be the cause of the reported lower growth rates of preterm infants fed with HoP-treated DHM, compared with the ones fed mother’s own milk (12). To overcome the disadvantages of this treatment, novel methods such as high-pressure processing (HPP), ultraviolet-C irradiation (UV-C), and thermoultrasonication (TUS) have been proposed as promising non-thermal alternatives to HoP (9, 13).

High-pressure processing is a mild food preservation method commonly applied in the food industry to guarantee the food safety of a product by microbial inactivation due to the high hydrostatic pressure (14, 15). UV irradiation is a non-thermal disinfection method, especially at wavelengths between 200 and 280 nm (UV-C) (16). This method effectively inactivates microbial contaminants by disrupting DNA transcription and replication, ultimately leading to cell death (16, 17). Ultrasonication (20–100 kHz) is a food preservation method that involves microbubble formation and their rapid collapse though inertial cavitation. The shock waves that are produced from this process, as well as the chemical changes induced by it, ultimately lead to bacterial cell death (9, 18). TUS, the process where ultrasonication is combined with mild heating, is considered more effective in microbial inactivation than ultrasonication alone (19). In addition to bacterial inactivation, all the aforementioned methods are able to batch process human milk, as would be required when applying these methodologies in a human milk bank.

When applied to DHM, these methods have shown promising results with regards to microbial inactivation and retention of DHM bioactive components (9, 20, 21). However, the number of studies evaluating UV-C or TUS as possible alternatives to HoP is still quite limited, while a large number of different pressure, time and temperature combinations have been applied for HPP to DHM, making direct comparison of those studies complicated (9).

The aim of this study was first to assess whether HPP, UV-C and TUS can achieve a 5-log_10_ bacterial reduction as found following HoP, which is a primary requirement for use of DHM. Secondly, we aimed to evaluate the effects of these methods on the DHM proteome in order to get a full overview of the changes caused, as well as on the concentration and bioactivity of IgA, LTF, LYZ, and BSSL, and compare them with HoP.

## Materials and Methods

### Milk Samples

**FIGURE 1:**
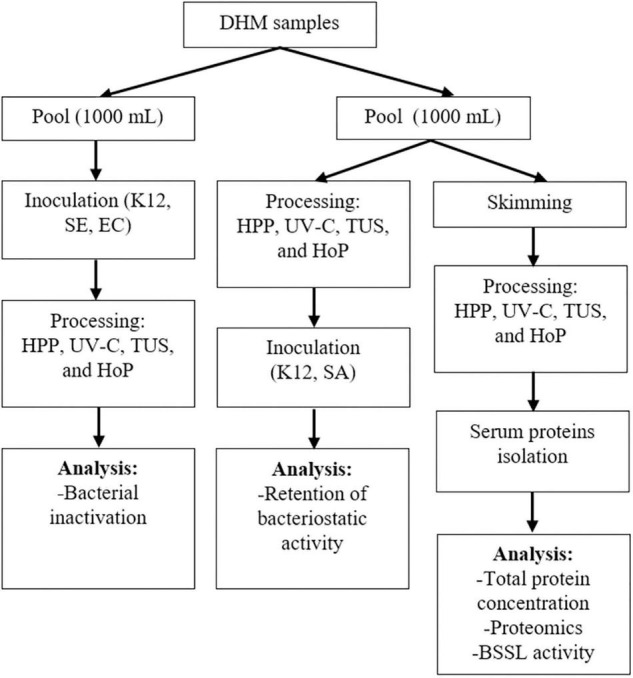
Schematic representation of the experimental approach used. K12, SE, and EC stand for *E. coli* K12, *S. epidermidis* and *E. cloacae*, respectively. Two independent experiments (biological replicates) were performed while all analyses were performed in technical duplicate for each sample.

The HM samples used in this study were provided by the Dutch Human Milk Bank (located at Amsterdam UMC, Amsterdam, Netherlands). Donor screening and milk collection was performed according to standardized procedures that comply to international guidelines (10). All donors signed informed consent before recruitment. Milk expression, collection, and transportation to the Dutch Human Milk Bank was performed as previously described (22). The samples were transported frozen (−20°C) to the human milk bank and were stored frozen at the same temperature, for a maximum of 3 months, until further processed. Before analysis, each donated sample was thawed overnight in a refrigerator at 4°C. Once thawed, the native microflora of the samples was assessed by pour or surface-plating of undiluted DHM (1 or 0.1 mL, respectively) in duplicate onto selective media (VRBGA, violet red bile glucose agar, CM0107B and MSA, mannitol salt agar, CM0085B, Thermo Fisher Scientific, Massachusetts, USA) and non-selective media (PCA, Plate Count Agar, CM0325, Thermo Fisher Scientific, MA, United States) followed by an incubation at 37°C for 24–48 h, while the remaining amount of each sample was again placed in the freezer (−20°C). Bacterial numbers were determined by colony counting (CFU/mL), and the samples with 1 log_10_ CFU/mL or less were selected. Next, the samples were again thawed overnight at 4°C, and milk from four different donors was pooled to ensure sufficient amounts of DHM for all treatments. One pool (four donors, 1,000 mL) was used to evaluate the inactivation of the selected bacterial strains and another pool (four donors, 1,000 mL) was used to evaluate the DHM proteome, the total protein content, the BSSL activity and the bacteriostatic properties. Aliquots for each treatment (HPP, UV-C, TUS, and HoP) and for the untreated milk (UN) that served as control were then created. After all treatments, the samples were cooled in an ice-water bath and all analyses were performed immediately. The analyses were performed as two independent experiments (biological replicates) in duplicate (technical replicates). The experimental approach used in this study is presented in Figure 1.

### Treatments

#### High-Pressure Processing

High pressure treatment was carried out in a pilot-scale equipment, custom made by Resato (1.6 L, Resato, Roden, Netherlands). The computer-controlled pressure build up was ∼30 MPa/s. The samples were subjected to three different pressures for various holding times; 400 MPa for 5, 10, and 30 min, 500 MPa for 1.5, 2 × 1.5, 3, and 5 min, 600 MPa for 1.5, 2 × 1.5, 3, and 5 min. For the treatments “2 × 1.5 min,” the process of pressure build up, holding time of 1.5 min and pressure release was carried out twice. This was done based on previous findings which demonstrated that two pressure treatments with 1.5 min holding time were more effective in microbial inactivation than one pressure treatment of 3 or 4 min at the same pressure (23). DHM samples (10 mL, 4°C) were packed into sterile pouches made of polyethylene. Two to four small sample pouches were packed in a larger pouch which was subsequently vacuumized at 95% vacuum. The larger pouches were then taped in a cylindrical holder that was placed in a sample holder as described previously (14). Samples were not preheated and tap water of 10°C was used at the start of the treatment as medium. The temperature increase during HPP treatment was described as follows (14): *T*_*increase*_/100*MPa* = 0.026×*T*_*initial*_+2.26, leading to a maximum sample temperature of around 18°C (after 600 MPa).

#### Ultraviolet-C Irradiation Treatment

The UV-C system was based on published literature (16), where a UV-C lamp (TUV PL-S 5W, UV-C radiation 1.1 W, Philips, Netherlands) was placed diagonally in a sterile beaker glass filled with 140 mL DHM. During treatment, the milk was stirred with a sterile 4 × 20 mm stirring rod at 500 rpm (IKA RH 2, Staufen, Germany). Samples (20 mL, 4°C) were taken at three different time points and were aliquoted for further analysis. The samples were exposed to three different UV-C dosages; 2,430, 3,645, and 4,863 J/L. The time needed to reach these dosages (5.15, 6.63, and 7.36 min, respectively) was calculated according to: $D\,o\,s\,a\,g\,e\,\left(\frac{J}{L}\right)=\frac{T\,i\,m\,e\,\left(s\right)\times U\,V-C\,P\,o\,w\,e\,r\,\left(W\right)}{V\,o\,l\,u\,m\,e\,\left(L\right)}$ (16). The temperature was controlled during the whole process with a temperature data logger (RS PRO 1384, RS Components B.V., Netherlands) and a maximum increase of 3°C was documented after a treatment of 4,863 J/L. This set-up was used in order to overcome the limited penetration of UV-C in milk (absorption coefficient of 300 cm^–1^ at 254 nm), by applying a turbulent flow (16).

#### Thermoultrasonication

A sonifier (Branson 450 Digital Sonifier^®^, Branson Ultrasonics Corporation, CT, United States) with a horn frequency of 20 kHz was outfitted with a sound enclosure (Branson Emerson Technologies, GmbH & Co, Germany), a microtip probe (length: 60 mm, diameter: 10 mm), and a circulating water bath. Samples (20 mL, 4°C) were placed into a sterile 80 mL glass beaker that was surrounded by circulating water of 40°C. The sonifier was operated in pulse mode, with a continuous pulse of 59.9 s followed by a short pause of 30 s. The samples were treated for 9 min (excluding pause time) at 40 W (1,080 kJ/L, 38% amplitude), for 6 min at 60W (1,080 kJ/L, 58% amplitude) or for 9 min at 60 W (1,620 kJ/L, 58% amplitude). The energy density (KJ/L) was calculated as power(W) x *treatmenttime*(*sec*)/*volume*(*mL*) (24). The temperature of the samples was recorded by a temperature data logger (RS PRO 1384, RS Components B.V., Netherlands). The maximum temperature increase was 20°C (maximum sample temperature, 59°C), after 9 min at 60 W.

#### Holder Pasteurization

Donor human milk (30 mL) was placed into a Greiner tube (50 mL) and was heated at 62.5°C for 30 min, in a shaking water bath (150 rpm). The sample was cooled in an ice-water bath immediately after treatment, until a temperature 4°C was reached. The time required for the temperature of the sample to reach the pasteurization temperature (62.5°C) was 25 min, while the cooling down time to 4°C was 15 min. A temperature data logger RS PRO 1384 (RS Components B.V., Netherlands) was used to monitor the temperatures during the whole process.

### Bacterial Inactivation

Bacterial species were selected based on their clinical relevance for DHM. Fresh cultures of *Enterobacter cloacae* (ATCC 13047, American Type Culture Collection, Manassas, VA, United States), *Escherichia coli* K12 (DSM 498, Deutsche Sammlung von Mikroorganismen und Zellkulturen, Braunschweig, Germany) and *Staphylococcus epidermidis* (ATCC 14990, American Type Culture Collection, Manassas, VA, United States) were prepared from frozen stocks in brain heart infusion broth (CM1135, Thermo Fisher Scientific, MA, United States) after an overnight incubation at 37°C. The bacterial pellets that were obtained after centrifugation at 4,000 × *g* for 10 min (Microcentrifuge 5890R, Eppendorf, Hamburg, Germany), were subsequently inoculated into DHM samples at a final concentration of 10^8^ CFU/mL and were then subjected to HPP, UV-C, TUS, or HoP treatment. Treated and untreated samples were next plated in duplicate onto VRBGA (*E. coli*, *E. cloacae*) and MSA (*S. epidermidis*) and were incubated overnight at optimal growth conditions. Untreated but inoculated samples with the three strains served as reference to verify the starting microbial concentration and to calculate bacterial reduction. The reduction in bacterial numbers was determined by colony counting (CFU/mL), with a detection limit of 0 log_10_ CFU/mL (*E. coli* and *E. cloacae* counts) and 1 log_10_ CFU/mL (*S. aureus* counts). Since the HPP unit used in this study is located in a food safe environment, inoculation with pathogenic strains was prohibited. Therefore, all the strains used in this study were biosafety level 1 strains. In addition, bacteria inactivation after HPP was tested only with the *E. cloacae* and *S. epidermidis* strains.

### Milk Serum Preparation and Total Protein Content

To obtain the native milk serum proteins, after all treatments, caseins and denatured proteins were removed. To do so, untreated samples (520 mL) were first centrifuged at 6,500 × *g* for 30 min at 4°C (with rotor 16.250, Avanti Centrifuge J-26 XP, Beckman Coulter, United States) to remove the fat. The skimmed samples were then treated with all methods as described above (sections 2.2.1–2.2.4), apart from one sample that remained untreated (control). Next, the pH of the skimmed samples was adjusted to 4.6 by the addition of 1 mol/L HCl under stirring, to precipitate the caseins and the denatured serum proteins (25). The samples were left for 30 min at 4°C to equilibrate and were subsequently ultracentrifuged at 100,000 × *g* for 90 min at 30°C (Optima L-80, Beckman Coulter, United States). Finally, the casein pellet was discarded, and the supernatant containing the native serum proteins was collected. The total native protein content was then assessed using the bicinchoninic acid (BCA) assay kit (Thermo Fisher Scientific, United States), according to the manufacturer’s instructions.

### Protein Quantification and Identification by Liquid Chromatography With Tandem Mass Spectrometry

#### Filter Aided Sample Preparation

The Filter Aided Sample Preparation (FASP)method was carried out as previously reported (26, 27). Briefly, milk serum samples were diluted with 100 mM Tris (pH 8.0) to a protein concentration of 1.0 μg/μL. The next steps included; DDT reduction (10 μL, 0.15 M), alkylation with 136 μL urea (8 M) in 100 mM Tris/HCl (0.1 M, pH 8.0) and 20 μL of acrylamide (0.2 M), placing the samples into ethanol washed Pall 3K omega filters (10–20 kDa cut off, OD003C34, Pall corporation, Port Washington, NY, United States) and centrifuging them at 14,000 × *g* for 30 min, adding 110 μL 50 mmol/L NH4HCO_3_ to the filters and centrifuging them again (14,000 × *g* for 30 min). The samples were then digested with 1 μL trypsin (0.5 μg/μL sequencing grade) in 100 μL of NH_4_HCO_3_ (0.05 M) and after an overnight incubation, they were centrifuged for 30 min at 14,000 × *g*. After the addition of 100 μL 1 mL/L HCOOH in water on the filters, another centrifugation followed (14,000 × *g* for 30 min). Finally, 3 μL of TFA (10% v/v) was added to the filtrate to adjust the pH of the samples to 3. All samples were stored at −20 °C prior to liquid chromatography with tandem mass spectrometry (LC-MS/MS) analysis.

#### LC-MS/MS Analysis

The LC-MS/MS analysis was performed as previously described (28). Briefly, the samples (5 μL) were injected onto a 0.10 × 250 mm ReproSil-Pur 120 C18-AQ 1.9 μm beads analytical column that was prepared in house, using pressure of 800 bar. The peptides were then eluted at a flow of 0.5 μL/min with an acetonitrile gradient. The gradient elution increased from 9 to 34% acetonitrile in water with 1 mL/L formic acid in 50 min. Next, an electrospray potential of 3.5 kV was applied straight to the eluent, through a needle that was equipped to the P777 Upchurch micro cross waste line. Using a Q Exactive HF-X quadrupole-Orbitrap mass spectrometer (Thermo Electron, San Jose, CA, United States), full scan Fourier Transform MS in positive mode between m/z 380 and 1400 were measured. MS/MS scans of the most abundant multiply-charged peaks were recorded in data-dependent mode. The obtained MS/MS data was analyzed using the Andromeda search engine of the MaxQuant software (v1.6.3.4). The Uniprot human protein database was used, together with a database containing the sequences of common contaminants (29). Protein identification and quantification was performed as previously described (30, 31). To calculate the false discovery rate (FDR), MaxQuant created a decoy database of reversed sequences. The FDR cut off used was 0.01. The required peptide length was set to at least seven amino acids, with a maximum of 2 missed cleavages allowed. Protein modifications were set for propionamide (C) (fixed) and oxidation (M) (variable). Contaminants (e.g., keratins, trypsin) were removed from the set of identified proteins, as well as the proteins that were detected in less than half of our samples.

### BSSL Activity

Bile salt-stimulated lipase activity was determined according to Krewinkel et al. (32), with minor modifications. This fluorometric assay allows the determination of lipase activity in DHM by utilizing the synthetic substrates 4-methylumbelliferyl butyrate (4-MUB) and 4-methylumbelliferyl laurate (4-MUL). Milk samples were first skimmed as described in section 2.4 and were then preincubated at 40°C for 3 min, under shaking (800 rpm) in a ThermoMixer (SmartBlock 1.5 mL, Eppendorf, Hamburg, Germany). The conversion of the added substrate was stopped by the addition of a stop solution containing GuHCl (8 M) and HCl (1 M) in water. Next, a neutralizing solution with Bis-tris (1 M), NaOH (0.85 M), and EDTA (0.25 M) in water was added to clarify the samples. The fluorescence was then measured with a fluorimeter (excitation 355 nm, emission 460 nm).

### DHM Bacteriostatic Capacity

To evaluate the effect of processing on the bacteriostatic capacity of DHM, the growth rate of *E. coli* and *S. aureus*, which are known to be sensitive to these proteins was characterized (5, 33, 34). Bacterial pellets of *E. coli* K12 (DSM 498) and *S. aureus* (ATCC6538, American Type Culture Collection, Manassas, VA, United States) were prepared as described in section 2.3 and were dissolved in peptone physiological salt solutions (PFZ; Tritium Microbiology, Netherlands). After determining the optical density (OD) with a spectrophotometer (Cary 50 UV–Visible Spectrophotometer, Agilent Technologies, United States) for bacterial culture standardization (35, 36), *E. coli* and *S. aureus* cultures were diluted and inoculated into untreated samples and samples that were previously treated with HPP, UV-C, TUS, and HoP, to a final concentration of around 10^3^ CFU/mL. This inoculation level was selected because higher levels may overcome the ability of the milk to inhibit the growth of *E. coli* and *S. aureus* (37). Next, the samples inoculated with *E. coli* and *S. aureus* were incubated at 37°C for 2 and 4 h, respectively. All samples were then plated in duplicate onto VRBGA (selective for *E. coli*) and MSA (selective for *S. aureus*) and the plates were subsequently incubated overnight at 37°C. The amount of inoculated DHM sample plated was 1 and 0.1 mL for VRBGA and MSA, respectively. The bacterial concentrations were then determined by colony counting (CFU/mL). The growth rate per hour was calculated as $l\,n\,\left(\frac{N_{t}}{N_{0}}\right)/\text{t}$, were N_*t*_ = bacterial counts after 2 or 4 h incubation, N_0_ = bacterial counts immediately after inoculation and t = incubation time.

### Data Analysis

Data analysis and visualization were performed using GraphPad Prism software 8.0 (GraphPad Inc., La Jolla, CA, United States). For multiple comparisons of means and to determine significant differences among the treatments, ANOVA and Tukey’s HSD for post-hoc testing were performed. Protein retentions (% compared to untreated) were calculated as the ratio of the concentration after each treatment to the concentration of untreated samples, multiplied by 100. Perseus software v.1.6.2.1 was used to analyze the intensity based absolute quantitation (iBAQ) values that were determined by MaxQuant. These values refer to the sum of all peptide peak intensities divided by the number of theoretically generated tryptic peptides and are considered as a good indicator for the absolute protein concentration (30). To indicate significant differences in the DHM proteome after the different treatments, student’s t-tests were performed in Perseus after imputation of missing values, using permutation-based false discovery rate (FDR) correction. The cluster analysis was performed and visualized with the circos.heatmap package in R version 4.1.2 (38) on the imputed log_10_ scaled IBAQ values. Pearson correlations were also calculated to determine the relationship between the bacterial growth rate and the retention of IgA, LTF, and LYZ. A correlation matrix was created using R version 3.4.0 (38). Significant differences in all analyses were indicated by a *p*-value < 0.05. Two independent experiments (biological replicates) were conducted and all analyses were performed in technical duplicates. Data are presented as mean ± standard deviation of the two independent experiments.

## Results

### Bacterial Inactivation

The bacterial count reductions after HPP, UV-C, TUS and HoP are presented in Table 1. A >7.8–log_10_ inactivation of *E. cloacae* and *S. epidermidis* was obtained after all the different HPP conditions tested. The same inactivation was obtained after HoP for all tested bacterial strains. A UV-C dosage of 4,863 J/L was the only UV-C dosage effective in causing a >5-log_10_ reduction of *E. cloacae, E. coli* K12 and *S. epidermidis* counts. All TUS conditions tested were able to achieve a >5-log_10_ reduction for all tested bacterial strains.

**TABLE 1:** Reduction in bacterial counts in HoP, HPP, UV-C, and TUS treated DHM samples.

Methods	Parameters	Log_10_ reduction (CFU/mL), mean ± SD		
		*Enterobacter cloacae*	*Staphylococcus epidermidis*	*E. coli K12*
HoP	>7.8 (below the detection limit)			
HPP	400 MPa, 5–30 min	>7.8 (below the detection limit)		ND*
	500 MPa, 1.5–5 min	>7.8 (below the detection limit)		
	600 MPa, 1.5–5 min	>7.8 (below the detection limit)		
UV-C	2,430 J/L	4.25 ± 0.1	5.00 ± 0.3	4. 36 ± 0.1
	3,645 J/L	4.64 ± 0.1	5.95 ± 0.1	5.30 ± 0.3
	4,863 J/L	5.78 ± 0.2	6.95 ± 0.4	6.92 ± 0.1
TUS	40 W for 9 min	6.31 ± 0.4	6.07 ± 0.3	6.40 ± 0.1
	60 W for 6 min	6.63 ± 0.6	6.50 ± 0.5	6.73 ± 0.3
	60 W for 9 min	6.52 ± 0.5	6.21 ± 0.2	6.79 ± 0.4

The results are presented as mean ± standard deviation of two independent experiments and all analyses were performed in technical duplicate. *ND, Not determined. The detection limit was 0 log_10_ CFU/mL for E. coli and E. cloacae and 1 log_10_ CFU/mL for S. aureus.

### Protein Damage

#### Native Milk Serum Protein Concentration

**FIGURE 2:**
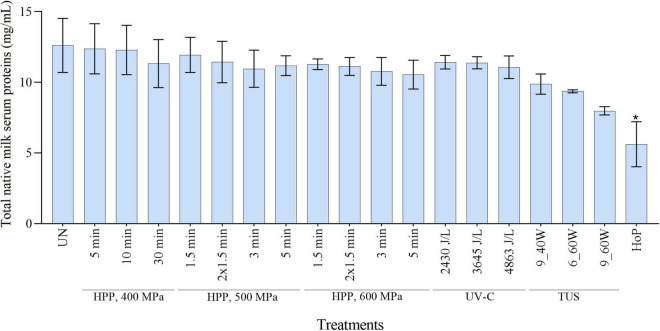
Native milk serum protein concentration as determined with a BCA assay. The results are presented as mean ± standard deviation of two independent experiments and all analyses were performed in technical duplicate. UN represents the untreated values. *Indicates significant differences to untreated samples (*p* < 0.05).

The total native milk serum protein concentration after HPP, UV-C, TUS, and HoP is shown in Figure 2. When compared to the untreated samples, a significant decrease in protein concentration was observed only after HoP (*p* < 0.05).

#### Effects of Processing on the DHM Proteome

**FIGURE 3:**
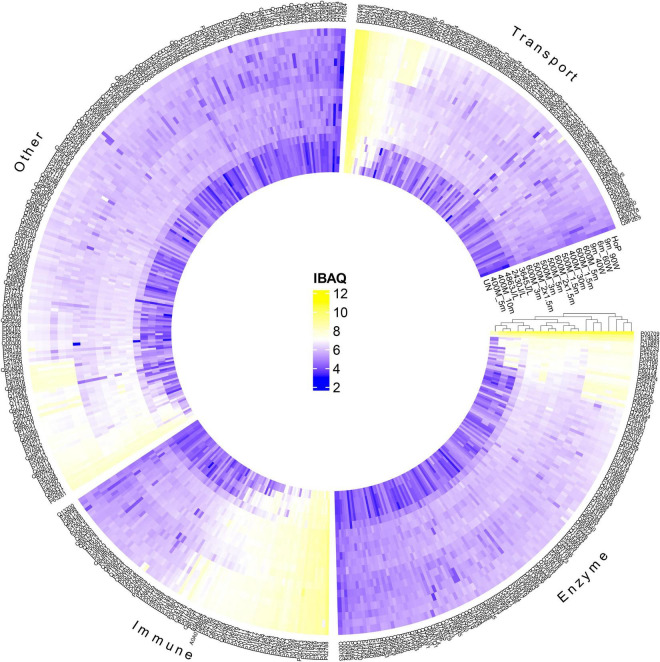
Hierarchical cluster analysis and heatmap showing the changes in the protein profile after HPP, UV-C, TUS, and HoP, based on iBAQ values (log_10_ scale from 2 to 12 according to color bar). Proteins are labeled by their UniProt ID. Functional categories (enzyme, immune, transport, and other) were based on GO annotation of biological function. Two independent experiments (biological replicates) were performed while all analyses were performed in technical duplicate for each sample. UN represents the untreated values. HPP; 400 MPa for 5, 10, and 30 min, 500 MPa for 1.5, 2 × 1.5, 3, and 5 min, 600 MPa for 1.5, 2 × 1.5, 3, and 5 min, UV-C; 2430J/L, 3645J/L, and 4863L/L,TUS; 9_40W, 6_60W, and 9_60W.

To further evaluate the effect of the different treatments on the native milk serum proteins, a detailed characterization of the DHM proteome was obtained, by means of LC-MS/MS. Next, a clustered heat map based on the obtained iBAQ values was created, for visualization of the protein profile of the different treated DHM samples (Figure 3). Samples with similar protein patterns are clustered together. The samples formed two main clusters; one that consists of the untreated, the HPP and the UV-C samples and one that includes the TUS and HoP samples. This clustering pattern indicates that HoP and TUS affect the DHM proteome the most, and similarly. In addition, the separation of the HPP treatments with the highest intensities (600MPa for 3 and 5 min) in the cluster with the other samples suggests that these most intense HPP treatments have a larger effect on the proteome compare to the less intense HPP and UV/C treatments.

#### Retention of IgA, LTF and LYZ After Processing

**FIGURE 4:**
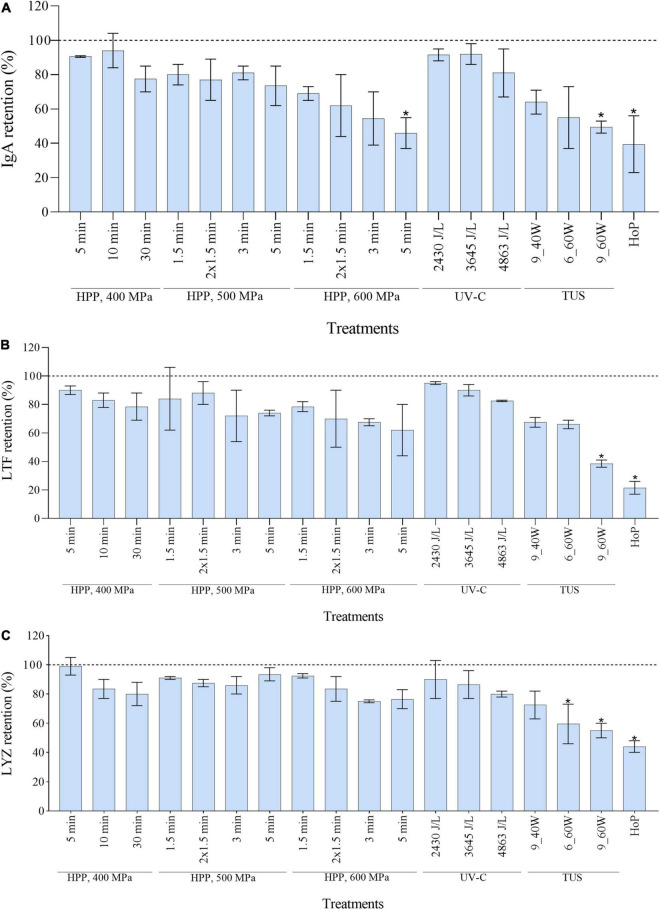
Effect of HPP, UV-C, TUS and HoP on the IgA **(A)**, LTF **(B)**, and LYZ **(C)** content. The retention values were calculated based on the iBAQ intensities obtained by LC-MS/MS analysis. Untreated values were set at 100% (dotted line). The results are presented as mean ± standard deviation of two independent experiments and all analyses were performed in technical duplicate.*Indicates significant differences to untreated samples (*p* < 0.05).

Of all the proteins analyzed by LC-MS/MS as shown in Figure 3, IgA, LTF, and LYZ are of importance due to their bacteriostatic activity. The retention values of the three proteins, as calculated from the LC-MS/MS data, were significantly reduced after HoP (*p* < 0.05), with only 40, 22, and 44% of IgA, LTF, and LYZ levels being retained after HoP, respectively (Figure 4). At the same time, none of the HPP treatments tested caused a significant reduction in LTF and LYZ levels. Furthermore, no IgA losses occurred after HPP at 400 and 500 MPa, regardless of the treatment time. When the pressure intensity increased (600 MPa), a treatment of 3 min caused a major decrease (55% IgA retention), although statistically non-significant, while a treatment of 5 min caused a statistically significant decrease (47% IgA retention). None of the applied UV-C dosages caused a significant reduction on the levels of the three proteins. The retentions of the three studied proteins showed a decreasing tendency with increasing TUS intensity and exposure time. After 6 min at 60 W, LYZ levels were significantly reduced, while after 9 min at 60 W, all three proteins were significantly reduced (*p* < 0.05).

#### BSSL Retention After Processing

**FIGURE 5:**
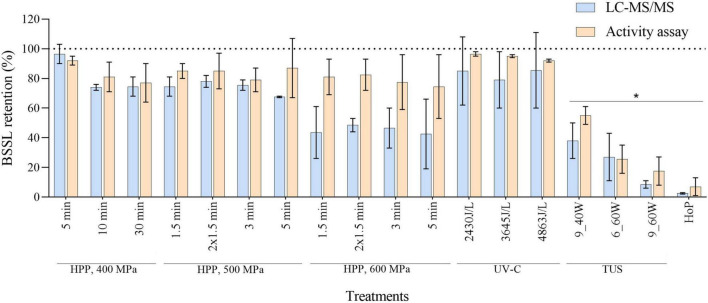
BSSL retention after HPP, UV-C, TUS, and HoP, based on iBAQ intensities and a lipase activity assay. Untreated values were set at 100% (dotted line). The results are presented as mean ± standard deviation of two independent experiments and all analyses were performed in technical duplicate.*Indicates significant differences to untreated samples (*p* < 0.05).

To evaluate whether the different methods affected the BSSL levels and activity, we first determined the BSSL retention, based on the LC-MS/MS results. Then, a specific lipase activity assay was used (as described in section 2.6), and the percentage of BSSL activity retained after the different treatments was compared to the LC-MS/MS values (Figure 5). Although the majority of the values obtained by the activity assay were higher than the LC-MS/MS values, no significant differences between the retention values from both analytical methods were observed (*p* > 0.05). After HoP, BSSL was almost completely diminished (LC-MS/MS, 3% and activity assay, 7%). On the contrary, the different HPP and UV-C treatments applied in this study did not lead to a significant decrease. However, BSSL retention decreased significantly after TUS, regardless of the intensity and the exposure time applied.

#### Retention of DHM Bacteriostatic Properties After Processing

**FIGURE 6:**
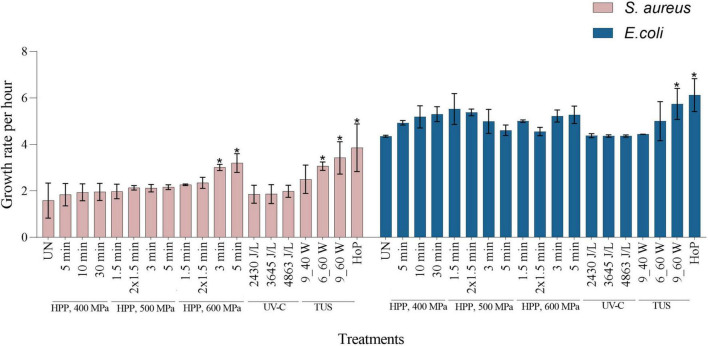
Growth rate per hour of *S. aureus* and *E. coli* in untreated, HPP, UV-C, TUS, and HoP DHM samples. The results are presented as mean ± standard deviation of two independent experiments and all analyses were performed in technical duplicate. UN represents the untreated values. *Indicates significant differences to untreated samples (*p* < 0.05).

To evaluate whether the bacteriostatic capacity of DHM was retained after the different treatments, the growth rate of *S. aureus* and *E. coli* was characterized, in both untreated and treated samples (Figure 6). Untreated samples showed the lowest bacterial growth rate, thus the highest inhibition rate for both strains (1.6 ± 0.75 and 4.4 ± 0.04-fold per hour, for *S. aureus* and *E. coli*, respectively). In contrast, the highest bacterial growth rate was observed after HoP (3.9 ± 1.02 and 6.1 ± 0.70-fold per hour, for *S. aureus* and *E. coli*, respectively, *p* < 0.05), which indicates a significant decrease in the DHM bacteriostatic capacity. When compared to the untreated samples, *E. coli* growth rate after HPP was not significantly different, while a significant increase in *S. aureus* growth rate was only observed at the highest intensities (3.0 ± 0.13 and 3.2 ± 0.40-fold per hour, after 600 MPa for 3 min and 600 MPa for 5min, respectively, *p* < 0.05). After UV-C, as well as after TUS for 9 min at 40 W, bacterial growth rates were not significantly different to those of untreated samples. The growth rate of *S. aureus* was significantly increased after 6 min at 60 W (3.0 ± 0.18-fold per hour), while after 9 min at the same intensity, the growth rates of both strains were significantly increased (*S. aureus*, 3.4 ± 0.69 and *E. coli*, 5.7 ± 0.66-fold per hour, *p* < 0.05).

**FIGURE 7:**
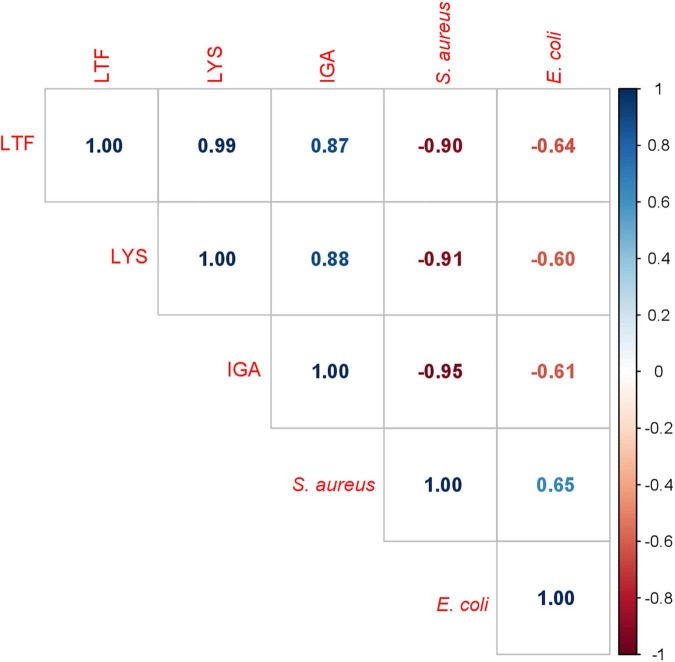
Correlation matrix of *S. aureus* and *E. coli* growth rates and IgA, LTF and LYZ iBAQ values. Each box contains an r value (Pearson correlation coefficient).

When the bacterial growth increased while the IgA, LTF, and/or LYZ levels decreased, a negative correlation could be expected between the *S. aureus* and *E. coli* growth rate and the retentions of these three antimicrobial proteins. To confirm this bacteriostatic activity, a correlation matrix was created (Figure 7). Figure 7 shows that *S. aureus* growth was strongly negatively correlated with the levels of these three antimicrobial proteins (IgA, r = −0.95, LTF, r = −0.90, and LYZ, r = −0.91, *p* < 0.05). Although the correlation between the inhibition of *E. coli* growth and the concentrations of these proteins was weaker, it was still significant (IgA, r = −0.61, LTF, r = −0.64, and LYZ, r = −0.64, *p* < 0.05).

## Discussion

The present study demonstrates that the tested HPP and UV-C conditions preserved the levels and functionality of key DHM bioactive components better than HoP, while at the same time ensured sufficient microbial inactivation. Although the tested TUS conditions resulted in similar bacterial inactivation, this method was generally less efficient in retaining the DHM bioactive components.

### Effects of Processing on Bacteria Inactivation

All different HPP intensity-time combinations were able to achieve a reduction >7.8-log_10_ CFU/mL of *E. cloacae* and *S. epidermidis* counts (Table 1), even at the lowest condition of 400 MPa for 5 min. Similarly, coliform and Enterobacteriaceae counts were reduced to undetectable levels after 5 min at pressures of 400–600 MPa (39, 40). Viazis et al. (41) also found a ≥6-log_10_ reduction of *E. coli* and *S. aureus* counts after 400 MPa for 30 min, while an 8-log_10_ reduction of *Listeria monocytogenes* and *Streptococcus agalactiae* counts was already achieved after ≤4 min at the same intensity. Although some *S. aureus* strains were found to be more pressure resistant, at higher pressures intensities significant reductions were achieved (500–600 MPa, or 400 MPa for 30 min for >5-log_10_ reduction) (41–43).

Only the highest UV-C dosage of 4,863 J/L was capable of achieving a >5-log_10_ CFU/mL reduction of *E. cloacae*, *S. epidermidis*, and *E. coli* K12 counts (Table 1). Christen et al. (16) showed similar reductions of *S. epidermidis*, *E. cloacae, Bacillus cereus*, and *E. coli* counts at 4,863 J/L, and according to Li et al. (44), the same dosage reduced DHM bacterial counts as effectively as HoP. Martysiak-Żurowska et al. (17) reported a 5-log_10_ reduction of *S. aureus* and *E. coli* K12 counts already at much lower dosages (400 and 700 J/L, respectively). The differences in the experimental set up (e.g., actual UV-C output power, milk flow around lamp, milk compositional differences) used in these studies may account for the observed variation.

All TUS treatments tested in this study achieved a >6-log_10_ reduction of *E. cloacae*, *S. epidermidis*, and *E. coli* K12 counts (Table 1). Similar results were observed by Czank et al. (45), who found that the decimal reduction time of *S. epidermidis* and *E. coli* K12 was 1.74 and 2.08 min, respectively after TUS at 60 W and 45°C.

In the current study, all HPP treatments, the highest UV-C dosage (4,863 J/L), and all three TUS treatments were capable of reducing bacterial counts in DHM samples >5-log_10_, thus meeting the requirements of the human milk banking guidelines.

### Effects of Processing on the DHM Proteome, IgA, LTF, LYZ, and BSSL Levels and Activity

In order to get a full overview of the impact of the different processing methods on the DHM proteome, the native milk serum protein levels were assessed by means of the BCA assay and LC-MS/MS. With regard to HoP, our results (Figures 2, 3) confirm the major decrease in native protein abundance (46). Moreover, the proteomic analysis of the differently treated DHM samples showed that HoP affected the native serum protein levels the most (Figure 3), an outcome that supported the results of the BCA assay (Figure 2). Of all the treatments tested in this study, HoP caused the highest reduction in IgA, LTF and LYZ levels (Figure 4), which is consistent with the losses previously reported (11, 47). As expected, the highest reduction in bacteriostatic capacity was also documented after HoP (Figure 6), which is in line with previous studies (5). These results can be attributed to the thermally induced denaturation and aggregation during HoP, which caused a loss in the functionality of these bioactive components (45). As BSSL is a heat-labile enzyme that inactivates at temperatures around 45°C (48), the complete loss of BSSL that was observed in this study was to be expected (11, 16, 21, 49).

We showed that HPP treatments at intensities of 400, 500 MPa and of short duration (<3 min) at 600 MPa, preserve the levels of the three main antimicrobial proteins in DHM (Figure 4). Furthermore, our proteomic analysis showed that HPP treatments at these intensities had only minimal effects on the levels of the native milk serum protein levels, while more intense conditions showed a larger change in these levels. The enhanced denaturation observed after HPP at 600 MPa at longer treatment times might be explained by the fact that HPP can cause native conformation unfolding and formation of inter/intra protein complexes, where these changes may only be reversible at lower treatment intensities (40, 50, 51). Moreover, increased protein denaturation has been observed at higher pressures and holding times, suggesting an effect of both pressure and holding time (52, 53). Other studies have also reported significant reductions in IgA levels after HPP at 600 MPa for >2.5 min (40, 54–57), while after treatment at 400–500 MPa, only 0–15% of IgA losses were documented (15, 39, 40). Our data showed that treatments of 400, 500, and 600 MPa retained LTF levels within a range of 62–90%, as previously described (52, 54, 58). In addition, none of the HPP treatments tested had an effect on LYZ levels, as well as on BSSL levels and activity, in line with previous studies (13, 15, 49, 56, 57, 59, 60). Since pressure and temperature have a synergistic effect on protein denaturation, the low initial temperature (4°C) and the limited temperature increase during HPP treatment (around 14°C at the most intense pressure of 600 MPa), may have additionally contributed to the improved protein retentions observed (61–63).

As a non-thermal method, UV-C does not inactivate pathogens by thermally-induced protein denaturation and aggregation, but by DNA disruption, that often results from pyrimidine dimerization (44). Hence, this method may effectively reduce bacterial counts in HM without causing detrimental losses of bioactive components (5, 44). All UV-C treatments in our study preserved both the levels and the bioactivity of IgA, LTF, and LYZ. In fact, the three antimicrobial proteins were retained within a range of 80–95% after UV-C treatment, while the bacteriostatic activity was similar to that observed in untreated HM (Figures 4, 6). The clustering pattern observed for the three dosages additionally suggests that the changes occurring in the DHM proteome after UV-C are minimal (Figure 3). Christen et al. (5) also reported retention of IgA, LTF, and LYZ within a range of 75–95% and no loss of bacteriostatic activity, after treatments of the same intensity. As it is possible that UV-C induces protein photo-oxidation (direct or indirect) the authors speculated that the reductions (∼25%) in LYZ levels at the highest dosage could be attributed to the fact that LYZ contains several amino acid residues that may absorb photons at this wavelength (5). With respect to the BSSL levels and activity, none of the dosages in this study showed a significant reduction compared to untreated milk (Figure 5), supporting previous findings (16, 44, 60).

After TUS at the highest ultrasound power (60 W) for the longest exposure time (9 min), the IgA, LTF, LYZ and BSSL levels and bioactivity retained were comparable to those after HoP (Figure 4). Similar reductions have been previously reported after 10 min at 60 W and 45°C (64). Treatments at 60 W for a shorter time (6 min) caused significant reductions in LYZ and BSSL levels and bioactivity, whereas at 40 W for 9 min, only BSSL was significantly reduced. Our findings suggest that at constant exposure times, higher ultrasound power will result in more protein damage. In addition, the differences observed when ultrasound energy was held constant (1,080 kJ/L after 9 min at 40 W or 6 min at 60 W), suggest that higher ultrasound power rather than the longer exposure time may lead to more protein damage. The impact of those treatments on the DHM proteome was confirmed by the hierarchical clustering analysis, that showed a similar pattern of protein damage to HoP-treated DHM (Fig. 3). These results could be further attributed to the temperature increase documented during such treatments, and to the denaturation that might be caused due to the shear effects generated during ultrasound cavitation (64, 65).

Specifically for BSSL, published reports have used both activity assays and quantification techniques (e.g., ELISA) to evaluate its retention (16, 66). Since the loss of protein as measured through LC-MS/MS approaches or ELISA assays is not necessarily correlated to loss of function (67), we compared the retention of the BSSL levels (LC-MS/MS) and the BSSL activity (activity assay) after the different treatments. Our findings suggest that although the activity assay produced higher values than LC-MS/MS values, no significant differences were observed between the results of the two methods.

Lastly, the correlations in this study between native IgA, LTF, and LYZ levels and the growth rate of bacteria sensitive to these proteins, suggest that these proteins may significantly contribute in limiting their growth (Figure 7). However, as DHM contains large numbers of antimicrobial components, the exact proportion of bacteriostatic activity attributed to those proteins is difficult to determine. These findings underline the importance of using complementary assays to determine protein levels and functionality, to accurately assess the effects of different processing methods. In this regard, many analytical techniques are available that can be used for future, more detailed characterization of such HM components and their functionality (28, 46, 68). In addition, the outcomes of our study suggest that certain proteins may be more sensitive to specific non-thermal treatments than others, due to the different underlying mechanisms of these treatments. Our proteomic analysis, for example, showed that even though TUS and high HPP intensities both cause protein damage, they cluster separately (Figure 3), indicating that different underlying mechanisms lead to a different profile of resulting protein damage. In summary, HPP at 400, 500, and 600 MPa for <3 min, as well as UV-C at 4,863 J/L, may be promising alternatives to HoP, when considering the sufficient microbial inactivation achieved and the improved outcomes on the preservation of important HM bioactive components.

## Conclusion

Although HoP is the method currently recommended for DHM processing, the results of the current study indicate that non-thermal methods such as HPP and UV-C may offer improved retention of key DHM bioactive components, while at the same time effectively reduce bacterial contaminants. These findings are of particular importance in the context of providing DHM to high-risk infants. However, before full-scale implementation of these technologies in a human milk bank setting, additional studies are needed to investigate both viral inactivation and the clinical significance of this study’s observations, especially with regards to growth rates and health status of infants fed DHM treated with HPP or UV-C.

## Data Availability Statement

The datasets presented in this study can be found in online repositories. The names of the repository/repositories and accession number(s) can be found below: ProteomeXchange Consortium via the PRIDE [Vizcaíno 2016] partner repository and PXD031216.

## Author Contributions

EK: conceptualization, methodology, investigation, and writing—original draft preparation. BS, RV, and JG: conceptualization, supervision, investigation, resources, and writing—reviewing and editing. SB: methodology, validation, and writing—reviewing and editing. RT and HB: methodology and writing—reviewing and editing. KH: conceptualization, supervision, investigation, resources, validation, and writing—reviewing and editing. All authors contributed to the article and approved the submitted version.

## Conflict of Interest

JG is the founder and director of the Dutch National Human Milk Bank and member of the Dutch National Health Council. BS is as Science Director of Human Milk Research & Analytical Sciences an employee of Danone Nutricia Research, Utrecht, Netherlands. The remaining authors declare that the research was conducted in the absence of any commercial or financial relationships that could be construed as a potential conflict of interest.

## Publisher’s Note

All claims expressed in this article are solely those of the authors and do not necessarily represent those of their affiliated organizations, or those of the publisher, the editors and the reviewers. Any product that may be evaluated in this article, or claim that may be made by its manufacturer, is not guaranteed or endorsed by the publisher.
